# A Bronze Age lip-paint from southeastern Iran

**DOI:** 10.1038/s41598-024-52490-w

**Published:** 2024-02-01

**Authors:** Nasir Eskandari, Eugenio De Carlo, Federico Zorzi, Stefano Dall’Acqua, Claudio Furlan, Gilberto Artioli, Massimo Vidale

**Affiliations:** 1 Faculty of Archaeology, University of Tehran, Enqelab-e Eslami Avenue, Tehran, Islamic Republic of Iran; 2 University Hospital Padua, Via Giustiniani, 2, 35128 Padua, Italy; 3 Department of Geosciences, University of Padua, Via G. Gradenigo 6, 35131 Padua, Italy; 4 Department of Pharmaceutical and Pharmacological Sciences, University of Padua, Via Marzolo 5, 35131 Padua, Italy; 5 Department of Cultural Heritage, University of Padua, Complesso del Liviano, 35139 Padua, Italy

**Keywords:** Drug discovery, Environmental social sciences, Risk factors, Chemistry, Materials science

## Abstract

A small chlorite vial, discovered among numerous artifacts looted and recovered in the Jiroft region of Kerman province, southeastern Iran, contains a deep red cosmetic preparation that is likely a lip-coloring paint or paste. Through analytical research involving XRD (X-ray diffraction), SEM–EDS (scanning electron microscopy-energy-dispersive spectroscopy), and HPLC–MS (high-performance liquid chromatography-mass spectrometry) analyses, the mineral components of the reddish substance were identified as hematite, darkened with manganite and braunite, and traces of galena and anglesite, mixed with vegetal waxes and other organic substances. The mixture, thus observed, bears a striking resemblance to the recipes of contemporary lipsticks. We also report the first radiocarbon date ever obtained from a Bronze age cosmetic in the ancient Near East: results place the pigment in the early 2nd millennium BCE, a date compatible with several mentions of the powerful eastern-iranian civilization of Marḫaši in coeval cuneiform texts of Mesopotamia, as well as with its currently emerging archaeological picture.

## Introduction

Late Chalcolithic to early Bronze age cosmetics (approximately 3500–1800 BCE) have been the subject of a growing number of analytical studies, which have unveiled previously unknown techniques of elaboration and chemical processing: a collateral branch of innovation in ancient metallurgy and organic chemistry so far largely neglected. In fact, in Egypt^1–5^, Anatolia^6^, Iran^7–9^ and Mesopotamia^10,11^, prehistoric stone flagons containing similar substances are common. Also, they have been unearthed in contemporary graves in southern Central Asia^12,13^, while lead-white, cerussite-based cosmetics were produced on a large scale using innovative techniques in late protohistoric northern China^14^. The apparent relative complexity of the early Iranian recipes may have been at least in part favoured by the greater complexity of the poly-metallic ores outcrops that distinguish large geological regions of the central Iranian Plateau^15^.

The frequent or systematic offering of cosmetic flagons to the deceased in burial contexts by several early urban, socially stratified societies across the time range of three millennia indicates that the artificial construction of social *personae* was an important aspect of interpersonal communication. It also suggests that such aesthetically constructed social *personae*, at least in some cases, were believed to survive (whatever the gender or identity was) the individuals' physical death.

Most of the cosmetic preparations so far analytically identified in the ancient Near East and Egypt were white or light-colored compounds (foundations or eye-shadows), to a great extent based on wet-processed lead, or black kohl eye-liners. However, analytically identified deep red pigments used for coloring lips have remained elusive and are still absent from existing inventories. When did people start to paint their lips red? Which pigments were first used for coloring human lips? In the same framework, was people aware of the potential dangers of direct lead ingestion from mouth?

**Figure 1:**
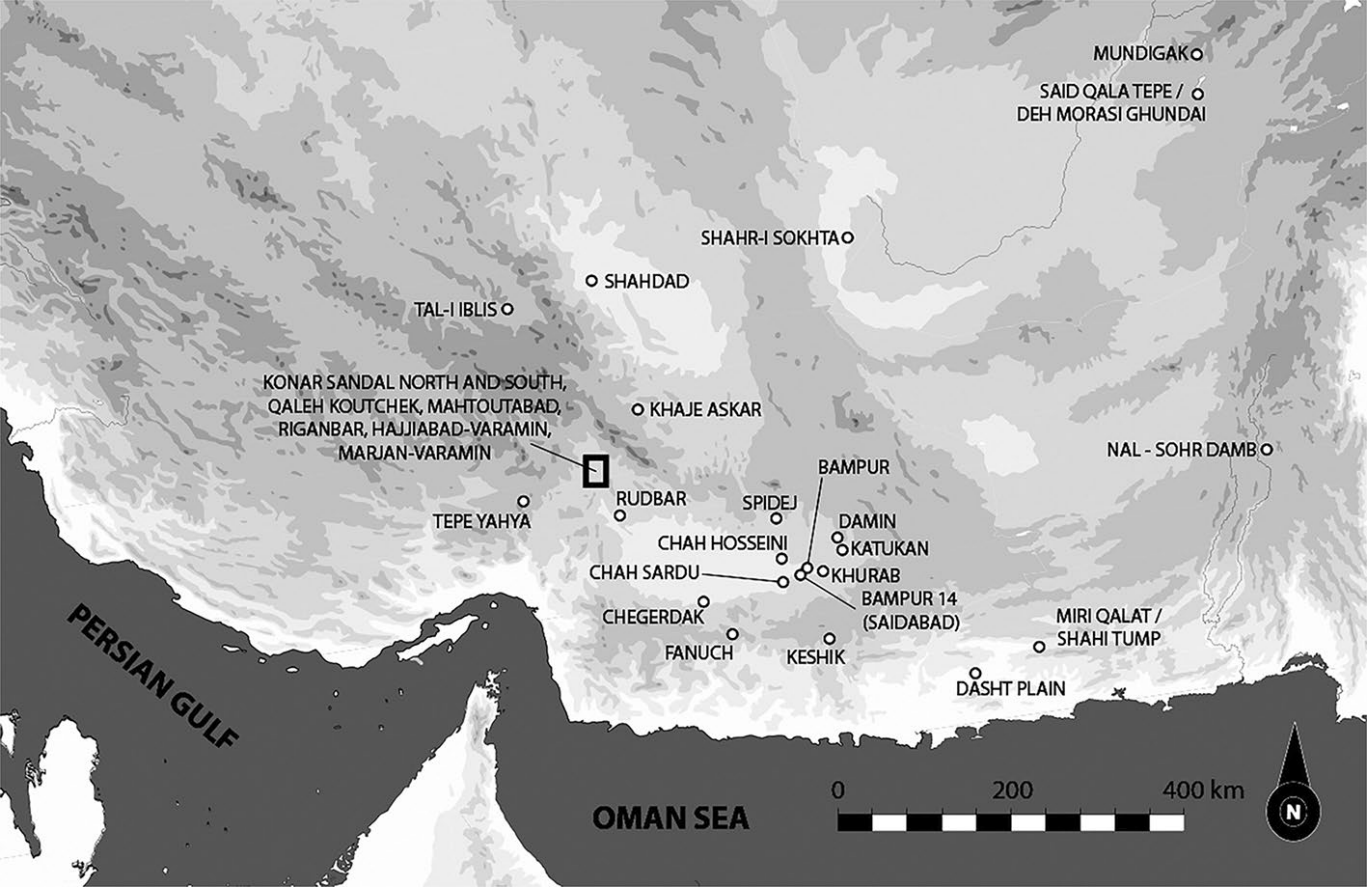
Map of the southeastern Iranian Plateau, including the relevant investigated Bronze age sites; the alluvial basin of the Halil Rud is here marked by a rectangle. From Eskandari N., Desset F., Hessari M., Shahsavari M., Shafiee M. and Vidale M. (2020) A late 4th to early 3rd millennium BC grave in Hajjiabad-Varamin (Jiroft, south- eastern Iran) : defining a new period of the Halil Rud valley protohistoric sequence, *Iranica Antiqua* 55, 1–48, figure no. 1 (with permission of the Author, F. Desset).

The Jiroft or Halil Rud civilization of ancient Iran (Fig. 1), now, seems to provide a first set of possible answers. The valley is formed by the tectonic collision forming the Zagros and causing subduction under the Makran with the Baluchistan volcanic formations. The drainage area of the Halil Rud esposes at west abundant metamorphic rocks (shale, marble, and large outcrops of chlorite-schists) while, at east, emerge diorites, granodiorites, granites, andesite, and volcanic ashes. To the south, the valley opens onto limestone, flysch and ryolithic massifs^16^.

As a consequence, this newly discovered civilization, probably the ancient polity of Marḫaši (thus in Sumerian, Paraḫšum in Akkadian) mentioned by coeval cuneiform texts of Mesopotamia^17^, was highly favoured in terms of direct access to a multifarious inventory of lithic resources.

Its archaeological remains were first discovered in 2001, when the Halil river (Kerman province, southeastern Iran) flooded its basin, hitting several graveyards of the 3rd millennium BCE. After rich burials came to surface, locals engaged in a prolonged season of open-air lootings, during which thousands of precious artifacts were robbed and lost to the antiques market^18–24^.

Many valuable artifacts in stone and copper were later recovered by Iranian security forces and eventually ended in the Jiroft archaeological Museum and other public collections at Kerman and Tehran. Although the precise provenience of the recovered finds will remain unknown, both the unmistakable style and the coherent assemblages confidently ascribe the greatest majority of the recovered lots to the local early Bronze age civilization, therefore to ancient Marḫaši.

## Material evidence

**Figure 2:**
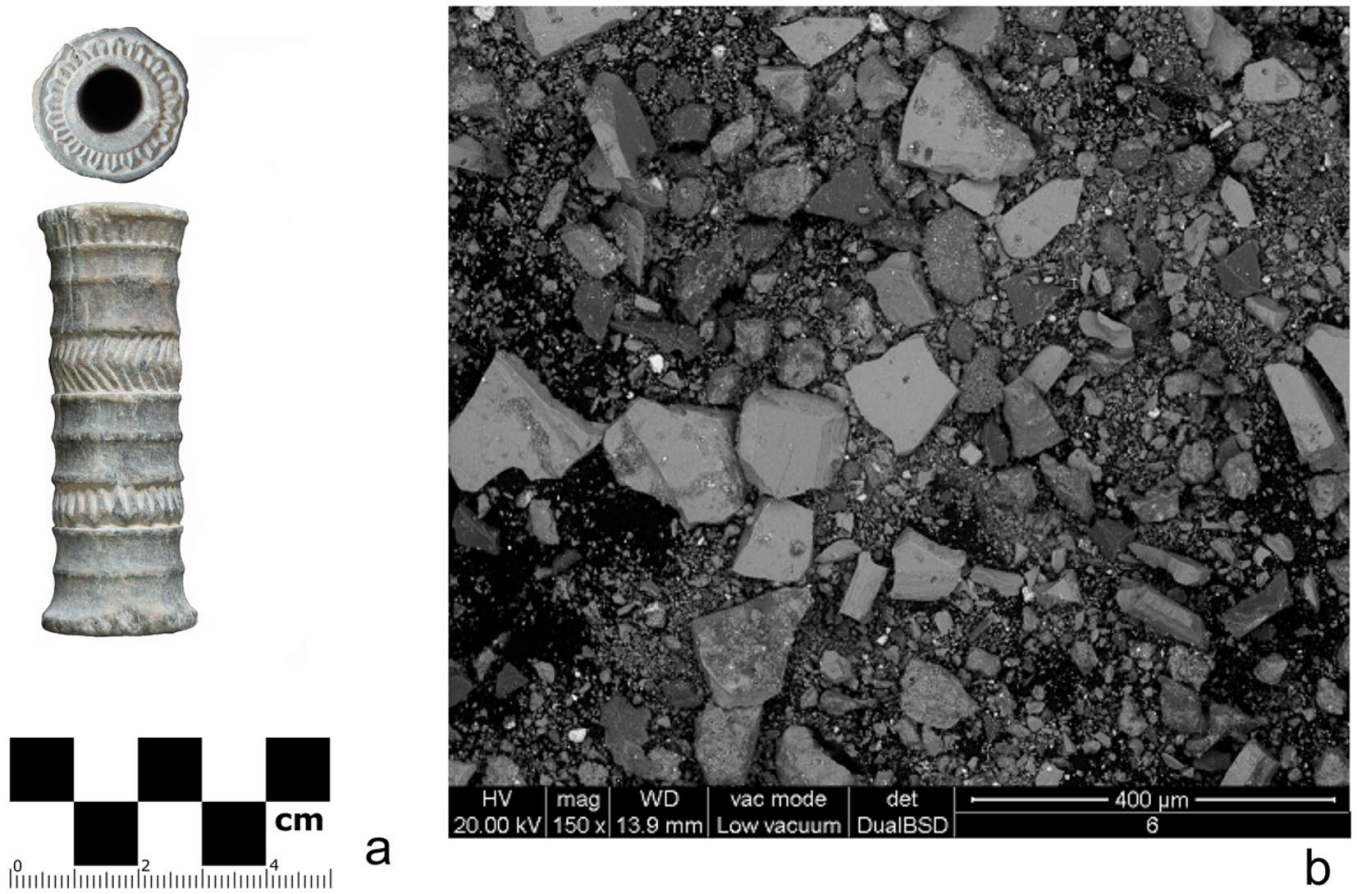
The chlorite-schist vial and its content. (**a**) the carved chlorite vial containing the cosmetic preparation. (**b**) a general ESEM view of the composition of the substance (EDAX analytical details in text) (images by M. Vidale, F. Zorzi).

This is the case of a small cylindrical vial here discussed (Fig. 2, left), at present kept with others at the Archaeological Museum of Jiroft. It is made of a greenish, compact chlorite-schist; its style is compatible with that of hundreds of other "Jiroft" chlorite artifacts, while its specific size and shape are distinct and unlike any other similar object currently known. The association between such a peculiar form and the quite unusual content discussed below potentially supports the idea that cosmetic products in ancient times were branded^25^, packaged and traded in standard types of containers with specific forms allowing for easy visual identification, as is the practice in contemporary parfumes and cosmetic industries—a trait of “modernity” certainly linked to the display of luxury and superior status by the emerging Bronze age elites.

The vial displays intricate craftsmanship, featuring fine incisions, and bears resemblance to a marsh cane segment, a type of container which was commonly used in various proto-urban societies in the Middle Asia and the Ancient Near East regions^26^. In this light, a more valuable semiprecious stone might have been chosen to make an object which, however, maintained the forms of a more common and much cheaper material—a kind of mimicry presumably desirable to preserve a set of tradional ideologic associations with the general domestic universe, while social distances between groups and families continued to grow.

The vial's slender shape and limited thickness suggest that it could have been conveniently held in one hand together with the handle of a copper/bronze mirror, leaving the other hand free to use a brush or another kind of applicator. In fact, a fragment of the famous Turin Papyrus 55001^27^ found at Deir el-Medina (Egypt) and dated to the New Kingdom, twelfth century BCE, shows a young woman who oints or perhaps paints her lips with a long brush or solid applicator in the right hand, while keeping at the same time with the left a large round mirror together with a thin, round-bottomed cylindrical cosmetic vial.

What was left of the contents was effortlessly extracted in the form of a loose, dark purple fine powder (Fig. 2, ESEM image at right). Soil burial contamination, in principle, cannot be excluded but in the light of subsequent observation at the optical microscope it looks minimal. Various analytical methods were employed to investigate its composition: Scanning Electron Microscopy with an Edax probe; semi-quantitative X-ray powder diffraction with RIR method; HPLC–MS analysis for the organic components; radiocarbon analysis for dating the pigment.

## Analytical results

**Figure 3:**
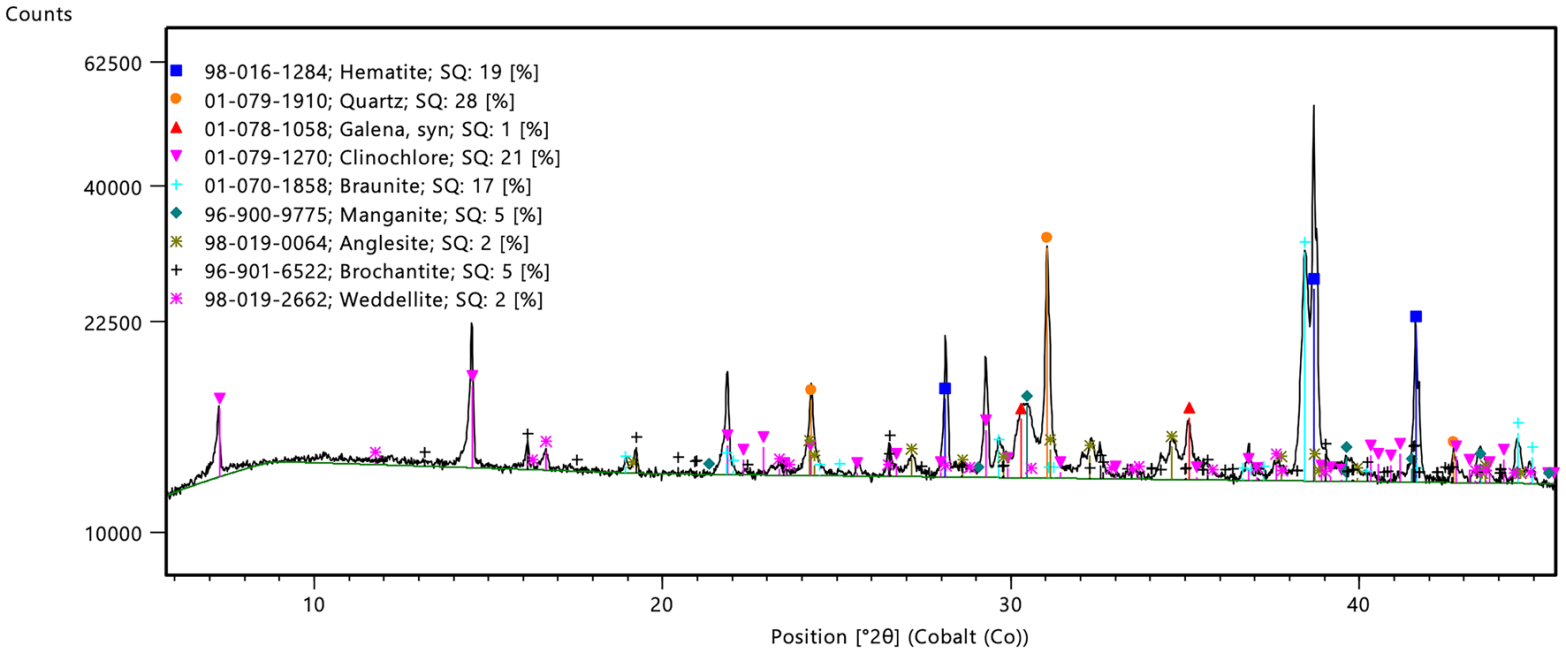
XRD spectrum: the peaks show hematite, quartz, galena, clinochlore, braunite, manganite, anglesite, brochantite and weddellite (F. Zorzi).

**Figure 4:**
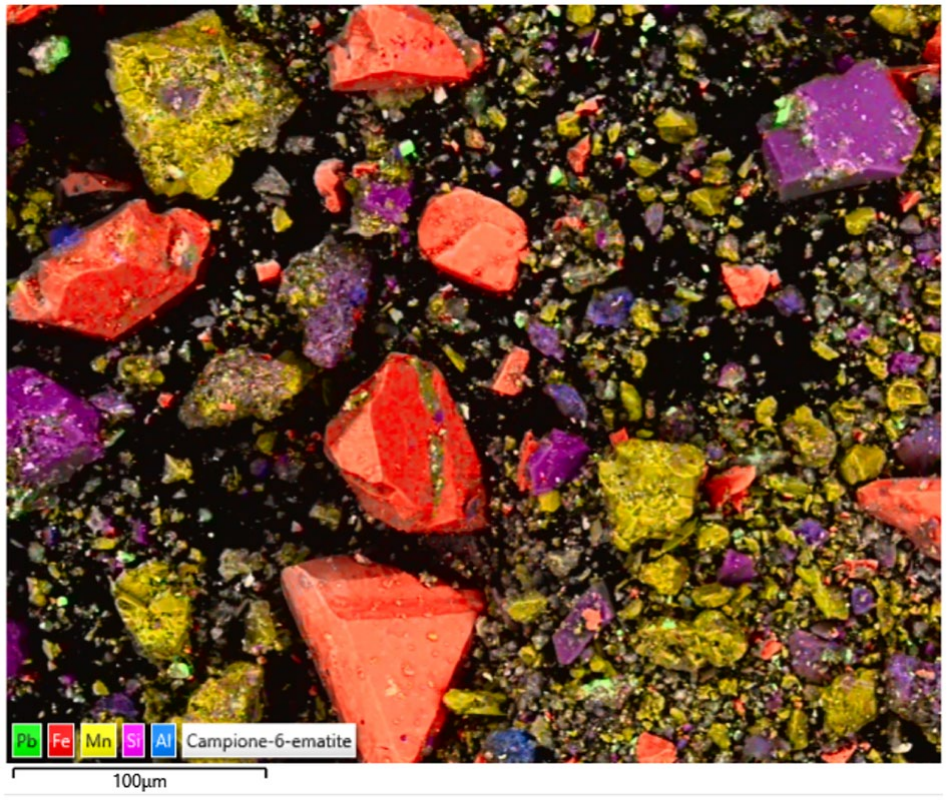
Mineralogical composition of the cosmetic preparation. Enhanced in false colours, red, micro-stratified fragmented sheets of hematite pinacoid forms; pseudo-octahedral crystals of braunite in yellow; fragmented (ground) quartz particles in pink. Rare cubic crystals of galena appear in green (F. Zorzi).

Eventually, XRD patterns (Fig. 3), aided by ESEM-microprobe inspection, showed that the cosmetic, in order of relative importance, was made of the following mineral components: hematite, Fe_2_O_3_—the dominant phase—in form of thin, micro-stratified pinacoid sheets; quartz, SiO_2_, as ground angular fragments with conchoidal fractures; braunite, Mn_2_ + Mn_3_ + 6(SiO_4_)O_8_, in pseudo-octahedral crystals, and manganite, MnO(OH), in prismatic forms; brochantite, Cu_4_(SO_4_)(OH)_6_; anglesite, PbSO_4_; and rare tiny crystals of galena, PbS. The respective amounts of lead minerals are very limited. These different phases are enhanced in false colours in Fig. 4.

The substance also contained some distorted and fractured vegetal fibers, seemingly different from the most usual taphonomical ingressions, possibly sources of aromatic components.

The high content of pure hematite was the source of an intense red color; in fact, hematite turns from black to red when ground in the size range of 0.48–0.11 mm^28^, compatible with that of the grains and crystals seen in Fig. 2b. Quartz particles (from ground sands or crystals) were added as a temper for the paste, or even as a shimmery-glittering agent, as encountered in other Bronze age cosmetics (although part of the quartz, as well as of the traces of clinochlore, could come from the inner walls of the decaying container).

In percentage, more than 80% of the analyzed sample is formed by minerals meant to produce a deep red. Braunite, a Mn brownish-black to steel gray silicate, manganite, a Mn oxide gray-black to black, plus isolated crystals of galena were all possible darkening agents with the exception of anglesite, a light-colored to colorless component (about 1%) that might be considered a secondary inclusion, possibly originating from the processed lead minerals. Brochantite could be due to the micro-localised corrosion of a copper applicator. In other Bronze age cosmetics from ancient Iran, weddellite—calcium oxalate—had been interpreted as a by-product of the use of urea for changing the colours of copper compounds^8^. In the present case, urea (if the source of calcium oxalate) could have been used for its moisturizing and exfoliating properties, as done for contemporary lip-sticks and other cosmetics^29^.

**Figure 5:**
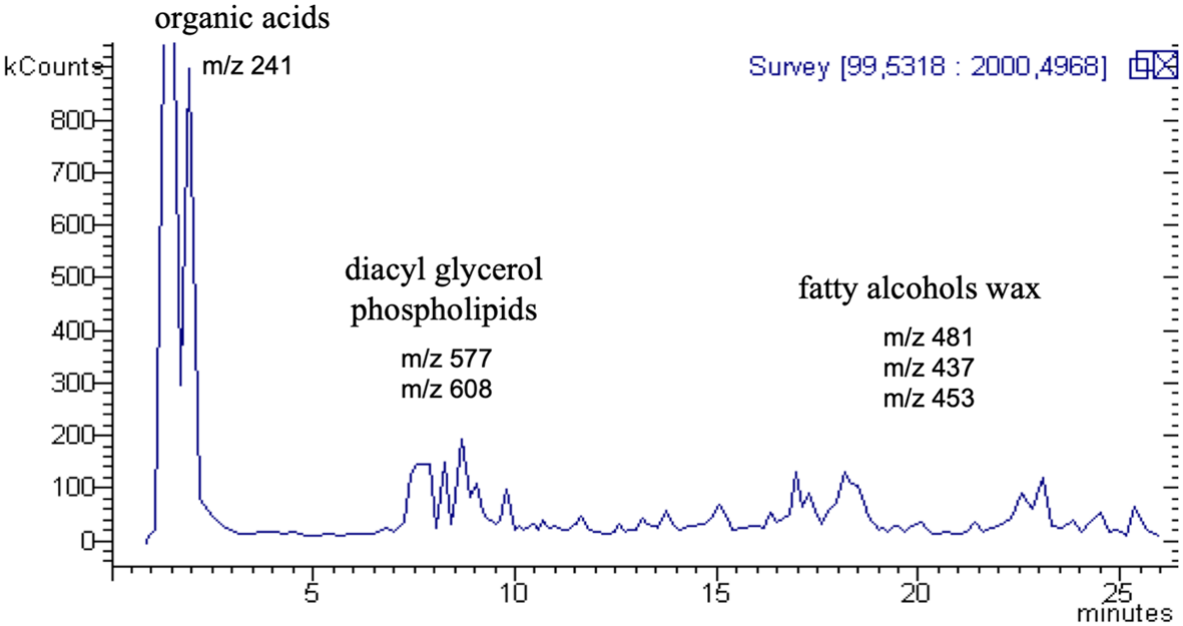
Chromatogram (kcounts vs. acquisition time) of the results of the HPLC–MS analysis of the cosmetic substance (S. Dall'Acqua).

HPLC–MS analysis (Fig. 5) detected 1-Hentriacontanol, Triacontanal e 1-Triacontanol, referable mainly to vegetable waxes. There are also phospholipid derivatives and derivatives of diacylglycerol with unsaturated fatty acids, presumably also of vegetable origin.

The included fibers could be linked to the other vegetal ingredients. Similar particles were also found in the cerussite/hydrocerussite-based white paste found at Shahdad^7^, another early Bronze age urban site of the region, datable to the mid fourth millennium BCE.

Both the intensity of the red coloring minerals and the waxy substances are, surprisingly enough, fully compatible with recipes for contemporary lipsticks. While Ancient Iranian foundations, eye shadows and rouges were mainly made of lead carbonates (cerussite/hydrocerussite) added with fine chromatically tuned colored preparations, the newly discovered cosmetic, as observed above, has minimal amounts of lead minerals. The contrast between the abundant use of lead-based substances apparently intended for the skin, and much less in this red paint, might suggest the makers of cosmetics were aware of the potential dangers of a direct oral lead ingestion.

## Radiocarbon dating and implications

**Figure 6:**
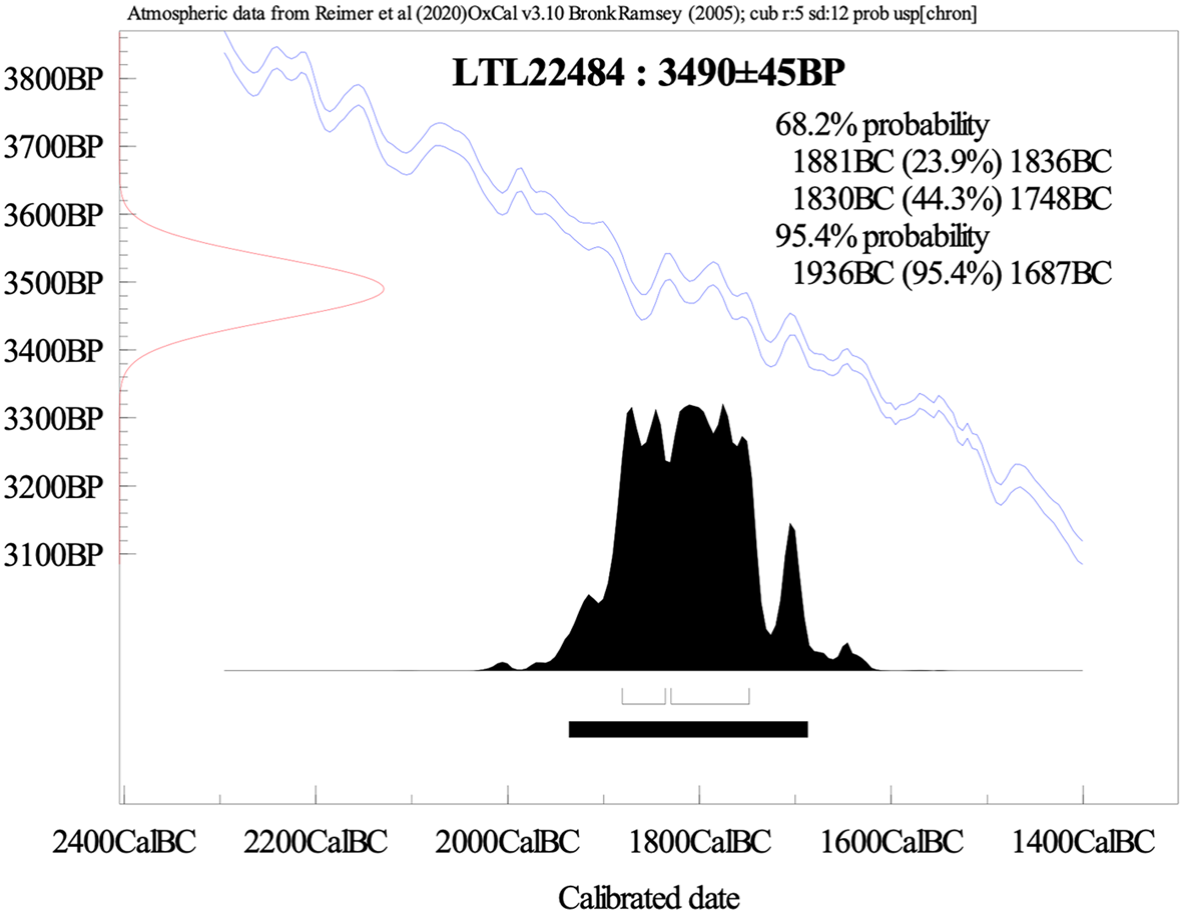
Calibration of conventional radiocarbon date of the sample of the red cosmetic (LTL22484), dated by the means of ^14^C with high-resolution mass spectrometry (AMS), at the CEDAD (Centro di Datazione e Diagnostica) of the University of Salento (Lecce, Italy).

We obtained a 14C date from the substance, indicating that its manufacture took place in the early 2nd millennium BCE. While the uncalibrated radiocarbonic age was 3490 ± 45 BP, specifically, the interval of 1936–1687 BCE (2σ, 95% probability) was determined as visible in Fig. 6. Given the absence of lead carbonates, it is believed that the primary sources of the isotopic signal are the organic components intentionally added to the original mixture. The relatively early date is far from surprising, considering the long-standing, well known technical and aesthetic tradition in cosmetology in ancient Iran^30^.

Currently, the recent 14C dates obtained from settlement contexts of the early city of Konar Sandal South (hereafter KSS), which is presumed to be the primary urban center in the Jiroft area during the 3rd millennium BCE, do not extend beyond the late twenty-fifth century BCE^20^. However, cuneiform records from the UR III dynasty (twenty-first century BCE) attest that ancient Marḫaši was a powerful political entity actively engaging with Mesopotamian powers at that time^31–33^. Moreover, recent intensive surveys conducted by one of the authors (N.E., ongoing research) around the main mound of site KSS support historical evidence, revealing a continuous sequence of settlements throughout the 2nd millennium BCE that, at present, remains to a great extent not fully documented and completely unexplored.

## Discussion

Ultimately, the deep red cosmetic is compatible with a lips coloring preparation—probably the earliest so far analytically reported—, and enriches the range of cosmetic practices within the chalcolithic-Bronze Age contexts under examination.In fact, specialised cosmetic technologies constituted a distinctive identitarian adaptation among the elites of Bronze Age southern Eurasia, intricately linked to the evolving social structures and their emerging performative functions, as discussed through actualistic approaches in contemporary literature^34,35^.

At present, given the generalized destruction of the Bronze age graveyards of the region, it is impossible to conclusively link the newly discovered substance to human remains, thus revealing agency, gender or sex-specific identities. In case this and other cosmetic substances, in certain periods and in certain contexts, should turn out to be consistently associated to female burials, one could explore the possibility that this gradually enhanced technology was also a function of increasing social stress to which women were exposed in times of fast social change^36^. On another level of interpretation, the evidence might also suggest that public images of female allure may have served both as a source of individual appeal and public authority in outstanding formal contexts of the earliest state-level societies of ancient Iran.

## Methods

Ultramicroscopic images of samples at different magnification were obtained and inorganic components (main, minor and trace elements) were identified with a low vacuum Environmental Scanning Electron Microscope (ESEM) system at the CEASC (Analysis Center and Certification Services of Padua University). A FEI Quanta 200 microscope operated in low vacuum, at 20 kV and 0.33 Torr pressure; samples were chemically analyzed with an EDAX probe and visually distinguished in false colours.

The mineralogical composition of samples was also studied by means of X-ray powder diffraction (XRD) experiments. The patterns were collected in the range 3–70° 2theta (400s per 0.017° step) using a PANalytical X'Pert Pro diffractometer equipped with a Co X-ray tube and a real-time multiple strip (RTMS) detector (X'Celerator). XRD semiquantitative analysis were performed with Panalytical High Score plus 4 using R.I.R. method implemented in the software.

Finally, we investigated the presence of organic contents, searching first of all for oils and waxes. For HPLC–MS analysis, the sample was weighted (1–10 mg) and added to a mixture formed by DMSO and methanol (50:50), vial was then sonicated for 15 min and then centrifuged at 13,000 rpm for 13 min. The supernatant was used for HPLC-DAD-ESI-MSn analysis. Ions were detected in positive and negative ion mode and MS fragmentation was obtained using the tdds (turbo depending data scanning) function of the instrument as well as HighRes mode acquisition allowed to obtain structural information on the compounds of the sample.

The radiocarbon date of the cosmetic was obtained at the CEDAD, center for radiocarbon dating by Accelerator Mass Spectrometry-AMS, of the University of Salento, Lecce, Italy. Macrocontaminants observed at the optical microscope were removed mechanically, and the selected fraction exposed to alternating acid–alkali-acid chemical attacks. The extracted material was then converted to carbon dioxide by combustion at 900 °C in an oxidizing environment, and then to graphite by reduction. The sample yielded 0.5 mg of graphite, which can be considered sufficient to obtain a reliable measurement with the AMS system. H2 was used as the reducing element and iron powder as the catalyst. Radiocarbon concentration was determined by comparing measured values of ^12^C and ^13^C currents, and ^14^C counts with values obtained from standard samples of C6 sucrose provided by the IAEA. Conventional radiocarbon dating was corrected for isotope fractionation effects both by measuring the δ^13^C term directly with the accelerator and by the background of the measurement. Samples of known concentration of oxalic acid provided by the National Institute of Standards and Technology (NIST) were used as a quality control. Both the scattering of the data around the mean value and the statistical error from counting ^14^C were taken into account in determining the experimental error in the radiocarbon date, then calibrated to calendar age using OxCal Ver. 3.10 software based on INTCAL20 atmospheric data.

## Data Availability

All analytical data gathered during the study are fully reported in this paper.
